# A functional evaluation of anti-fatigue and exercise performance improvement following vitamin B complex supplementation in healthy humans, a randomized double-blind trial

**DOI:** 10.7150/ijms.86738

**Published:** 2023-08-15

**Authors:** Mon-Chien Lee, Yi-Ju Hsu, Sih-Yu Shen, Chin-Shan Ho, Chi-Chang Huang

**Affiliations:** 1 Graduate Institute of Sports Science, National Taiwan Sport University, Taoyuan City 333325, Taiwan.; 2 Graduate Institute of Applied Science and Engineering, Fu-Jen Catholic University, New Taipei City, 242062, Taiwan.; 3 Tajen University, Pingtung 907101, Taiwan.

**Keywords:** B vitamins, exercise performance, antifatigue, endurance, health

## Abstract

B vitamins play a crucial role in maintaining fundamental cellular functions and various essential metabolic pathways in the body. Although they do not directly provide energy, each B vitamin acts as a cofactor in energy metabolism processes. Based on the evidence presented above, we hypothesized that a 28-day supplementation of vitamin B would enhance physical performance and reduce physical fatigue. The objective of this study was to evaluate the anti-fatigue effect of vitamin B supplementation, specifically vitamin B1, B2, B6, and B12, and its potential to improve exercise performance. We employed a randomized double-blind crossover design with a 28-day supplementation period. Sixteen male and sixteen female subjects, aged 20-30 years, were divided into two groups: the placebo group (n=16, equal gender distribution) and the Ex PLUS^®^ group (n=16, equal gender distribution). The participants received either placebo or Ex PLUS^®^ (one tablet per day) for 28 consecutive days. Following the intervention, there was a 14-day wash-out period during which the subjects did not receive any further interventions. After supplementation with Ex PLUS^®^, we found a significant increase in the running time by 1.26-fold (*p <* 0.05) to exhaustion compared to that before supplementation and that in the placebo group. In addition, the Ex PLUS^®^ supplementation group presented significantly reduced blood lactate and blood ammonia concentrations during exercise and at rest after exercise compared with placebo (*p* < 0.05). In conclusion, 28 consecutive days of vitamin B complex (Ex PLUS^®^) supplementation significantly improved exercise endurance performance and reduced exercise fatigue biochemical metabolites in not athletes. In addition, it does not cause adverse effects in humans when taken at appropriate doses.

## Introduction

Fatigue, defined as the inability to maintain power output and strength, is a symptom or comorbidity of neurological disorders 1. An important feature of fatigue is the "feeling of exhaustion," the mental or physical exhaustion that occurs when the demands of the brain or muscles cannot be met 2. Among them, physiological fatigue is further divided into central fatigue and peripheral fatigue, which are mainly caused by excessive physical load, insufficient rest, and mental stress 3. A previous study found that more than 50% of people feel fatigued, and more than one-third of them explicitly believe that they are affected by fatigue, which severely reduces their quality of life and productivity 4. However, it is also a physiological mechanism of self-protection after the body reaches a certain level of activity, which can prevent the occurrence of life-threatening excessive functional failure 5. During exercise, with increasing exercise time or intensity, a large amount of energy is consumed. If the original supply cannot be maintained continuously, the glycogen in the liver and muscle will be broken down into glucose, then further metabolized to meet higher energy needs 6. When metabolites such as lactic acid, blood ammonia, and blood urea nitrogen accumulate too much, resulting in an imbalance of pH and osmotic pressure, energy supply cannot be maintained, resulting in muscle fatigue and reduced exercise performance 7. Therefore, it is necessary to adopt appropriate strategies to improve or delay the generation of fatigue, among which a strategy through diet or nutritional supplementation is one of the most direct methods 8.

In addition to specific efficacy properties, many foods contain essential nutrients, including vitamins and minerals, which play an important role in maintaining essential cellular functions and various essential metabolic pathways 9, including involvement in energy production, DNA synthesis, oxygen transport, and metabolism 2. Among them, the vitamins B are a group of eight water-soluble vitamins, all of which, except folic acid, are involved in at least one step of the intracellular energy production system 10. Vitamin B acts as a coenzyme in metabolic and anabolic enzymatic reactions and is a cofactor for many essential enzymes involved in RNA and DNA biosynthesis 11. An adequate supply of each vitamin B is necessary for the proper functioning of the energy production system, but each vitamin B has different pathways involved in energy metabolism. A shortage of any of these vitamins can limit the rate of energy production and can have serious metabolic and health consequences 12.

Vitamin B1 (thiamine) is rapidly absorbed by the small intestine into three phosphorylated forms: thiamine monophosphate (TMP), thiamine pyrophosphate (TPP), and thiamine triphosphate (TTP) 13. TPP is involved in protein, lipid, and carbohydrate metabolism to produce dehydrogenase reactions, resulting in the decarboxylation of pyruvate and branched-chain amino acids (BCAA) to form acetyl-CoA 14. Vitamin B2 (riboflavin) is a component of the coenzymes flavin adenine dinucleotide (FAD) and flavin mononucleotide (FMN), redox cofactors of several dehydrogenases involved in energy metabolism, redox balance, and other cell regulatory processes 15. Vitamin B6 (pyridoxine) plays an important role in the metabolism of fatty acids, carbohydrates and amino acids and plays a key role in the energy production of the citric acid cycle 16. It is involved in amino acid metabolism, one-carbon reactions, glycogenolysis and gluconeogenesis, heme synthesis, and tryptophan formation from niacin, as well as lipid metabolism and hormonal action, and it provides additional glucose when needed^2^. Vitamin B12 (cobalamin) is a general term for a group of compounds called corrinoids, coenzymes involved in the metabolism of every cell in the human body, especially affecting the synthesis and regulation of DNA but also affecting fatty acid metabolism and amino acid metabolism 16, 17. The above four vitamins B (B1, B2, B6, B12) are mainly involved in energy metabolism and utilization, and they are also the main vitamin B types commonly used as dietary supplements 18.

While food intake is the main source of energy, vitamins B act as catalysts, helping to convert energy into better uses to more efficiently supply what the body needs 19. Therefore, to a certain extent, taking an appropriate amount of vitamin B complex can also be regarded as a sports nutrition supplement, thereby improving exercise performance. However, no prior studies have examined the benefits of vitamin B supplementation on exercise performance alone. Therefore, in this trial, we aimed to evaluate the benefits of vitamin B complex supplementation in enhanced exercise performance, delayed fatigue, and physiological adaptation in humans.

## Materials and methods

### Vitamin B complex and placebo preparation

The supplement in this study was provided by Prince Pharmaceutical Co. Ltd. (New Taipei City, Taiwan). One vitamin B complex tablet (Ex PLUS^®^) contains vitamin B1 (33.6 mg), vitamin B2 (10 mg), vitamin B6 (50 mg), vitamin B12 (750 µg), vitamin E (16.8 mg), inositol (20 mg), calcium (18.9 mg), and taurine (20 mg). The placebo had the same color and size but did not contain the above active ingredients.

### Participants

**Figure 1:**
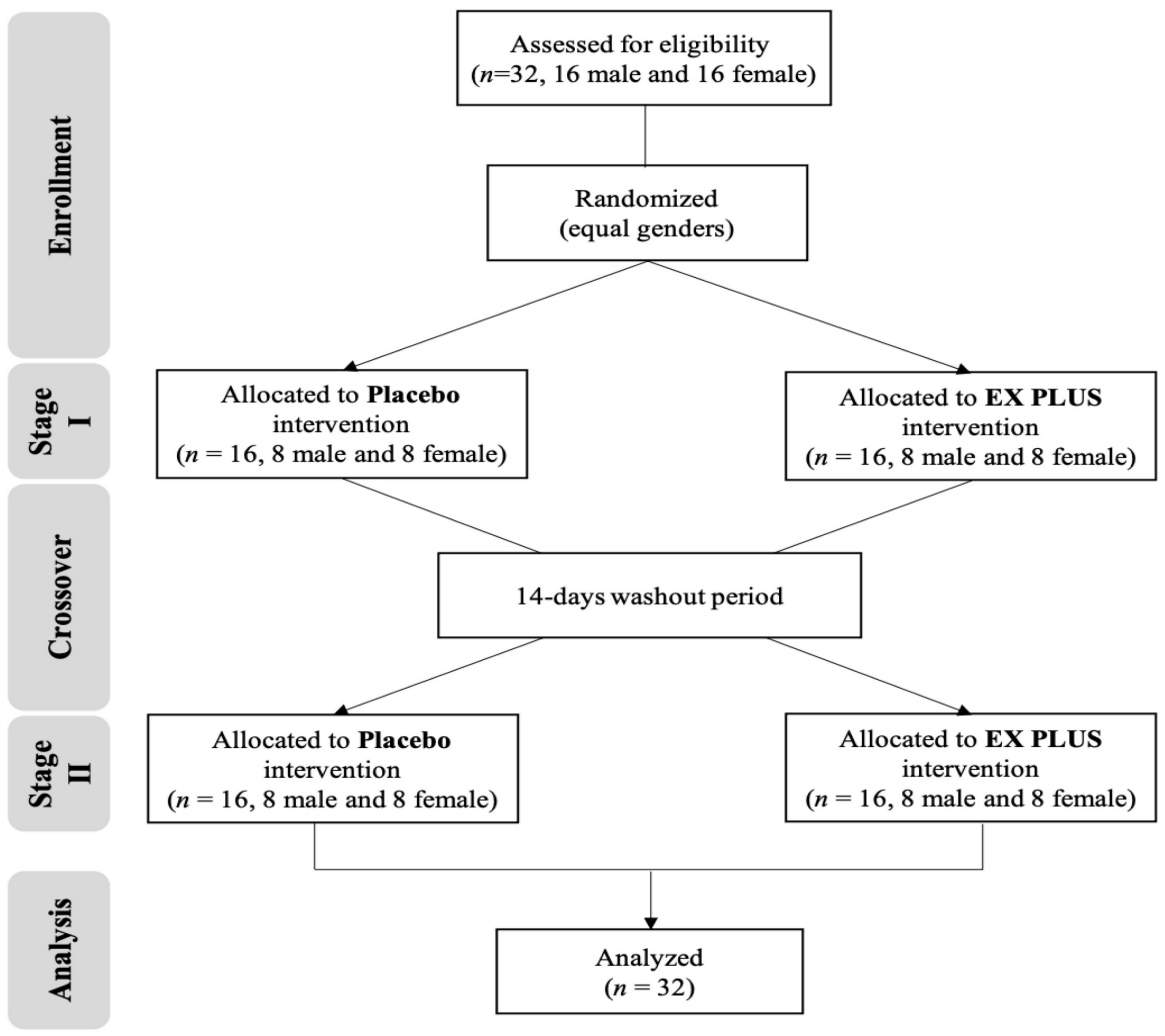
Participant flow chart.

This study was conducted in accordance with the Declaration of Helsinki and was approved and reviewed by the Institutional Review Board of Landseed International Hospital (Taoyuan, Taiwan; LSHIRB number 20-037-A2). The trial is first registered with clinicaltrials.gov as NCT05586295 (09/12/2022). Sixteen male and sixteen female healthy adult non-athletes aged 20-30 were included. Exclusions were made based on smoking, cardiovascular disease, hypertension, body mass index (BMI) >27, metabolic disease, asthma and sports injury (nerve, muscle, bone). After a detailed explanation of all the risks and benefits of the experimental procedure, the consenting participants signed the informed consent form in person before starting the experiment. Subjects were required to cooperate by maintaining a normal diet during the experiment and not taking nutritional supplements such as alcohol or vitamin-B-related products. The participant flow chart was shown on **Figure 1**, and subjects' basic information as shown on **Table 1**.

### Experimental design

Based on previous testing periods of animal and human B-vitamin supplementation, a duration of 28 consecutive days was chosen as the supplementation period 14, 20. For this trial, we used a randomized double-blind crossover design, the blinding was assisted by Prince Pharmaceutical Co., Ltd. (New Taipei City, Taiwan), and the blinding was lifted after the experiment was completed. Subjects were randomly assigned to a placebo or Ex PLUS^®^ group (n=16/group, equal genders) by random selection and supplemented with placebo or Ex PLUS^®^ (one tablet per day) for 28 consecutive days. Following the intervention, there was a 14-day wash-out period during which subjects received no further intervention. This was followed by a second 28-day replacement therapy intervention cycle (subjects who received placebo during the first cycle received Ex PLUS^®^ and vice versa). All subjects were supplemented only during the experiment and did not participate in any exercise training.

**Figure 2:**
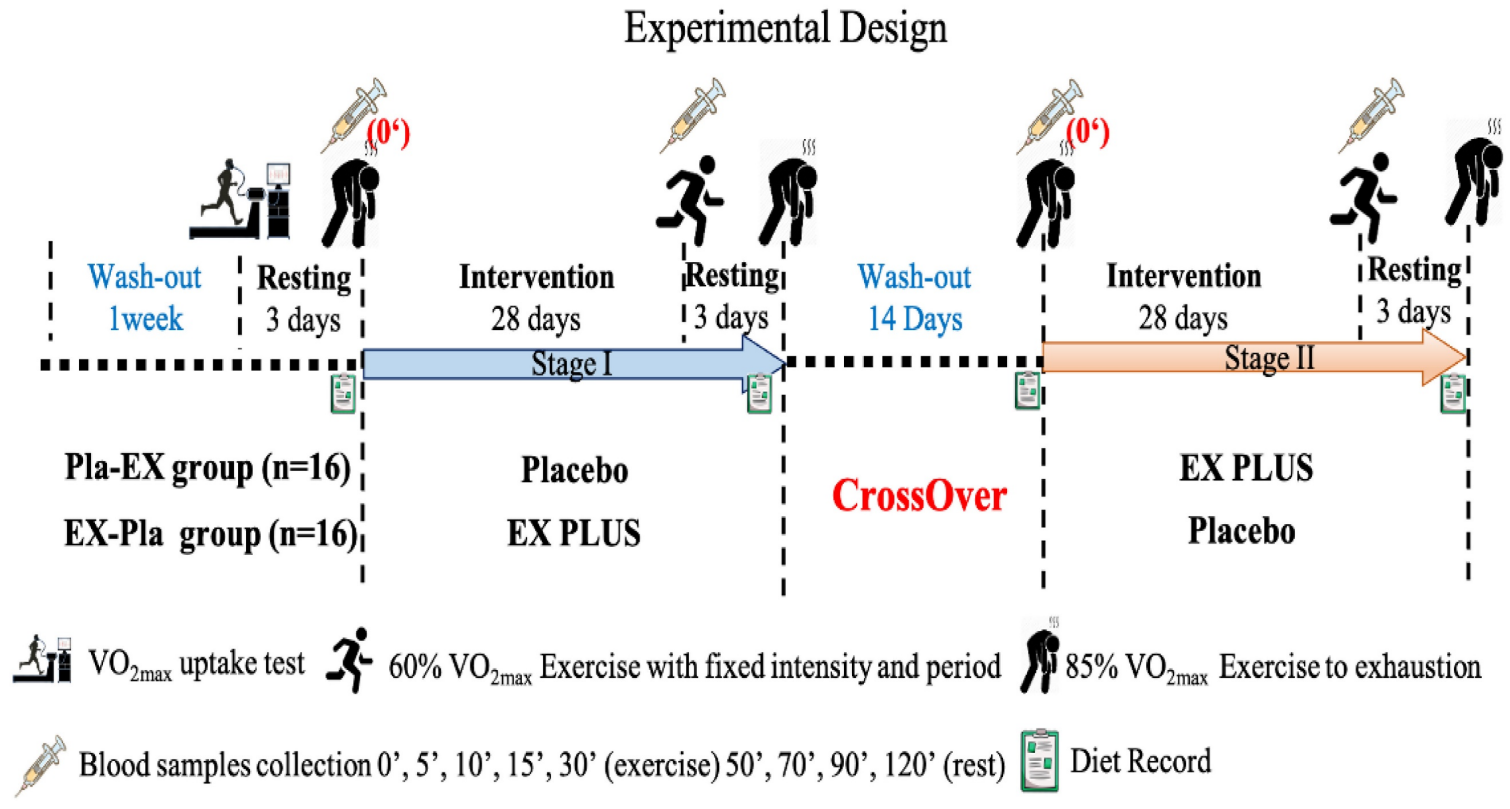
Experimental design.

Before each phase of intervention, we measured the subjects' body composition, common blood biochemical parameters, and exercise tolerance. After consecutive 28 days of supplementation, we re-measured the subjects' exercise fatigue biochemical values, exercise endurance performance, and body composition. In addition, we asked all subjects to record their diet before and after the intervention, and a professional nutritionist analyzed the daily nutrient intake to ensure that the relevant sports performance was not affected by diet (**Figure 2**).

### Maximal oxygen uptake (VO2max)

VO_2max_ was used as a reference for exercise fatigue detection and exercise endurance intensity setting. We performed VO_2max_ measurements according to Bruce's protocol and as previously described 21, 22, using a treadmill (Pulsar, h/p/cosmos, Nussdorf-Traunstein, Germany) and an automatic breathing analyzer (Vmax 29c, Sensor Medics, Yorba Linda, CA, USA), and monitored heart rate (HR) using a polar heart rate device (Polar Electro Oy, Kempele, Finland). The treadmill started at 7.2 km/h and increased by 1.8 km/h every 2 minutes until exhaustion. When the HR reached the maximum (maximum heart rate = 220-age), the respiratory exchange rate exceeded 1.10 (the volume ratio of carbon dioxide produced to oxygen consumed, VCO_2_/VO_2_), and the rating of perceived exertion (RPE) reached 10, a subject was determined to be exhausted. We then averaged the three highest VO_2max_ values in the test for the subjects to obtain VO_2max_ values.

### Endurance performance test and exercise fatigue-related indicators

According to the heart rate and speed recorded during the maximum oxygen uptake test, a regression calculation is performed to obtain the heart rate and speed corresponding to 60% and 85% of the maximum oxygen uptake, and the speed is adjusted according to the heart rate value. The detailed formula for intensity adjustment was based on a previous study 23. Before the formal test, according to each subject's personal 60% VO_2max_ intensity, after a 5-minute warm-up on the treadmill, we set the intensity to a maximum of 85% VO_2max_ to start the test. Subjects were instructed to run to exhaustion on a treadmill, and the time was recorded as their individual endurance performance. We monitored the subjects' physical condition every 2 minutes by heart rate.

On the other hand, to assess changes in indicators of fatigue, all subjects were asked to fast for at least 8 hours before the test. On the test day, we set a personal 60% VO_2max_ intensity for each subject to run for 30 minutes and rest for 90 minutes, during which blood was collected at baseline; at exercise periods 5 (E5), 10 (E10), 15 (E15), and 30 (E30) minutes; and at 20 (R20), 40 (R40), 60 (R60), and 90 (R90) minutes of rest after the 30 minutes of exercise. All collected blood was centrifuged to obtain serum and analyzed for fatigue indicators, including lactate, blood ammonia (NH_3_), glucose, and creatine kinase (CK), using a Hitachi 7060 autoanalyzer (Hitachi 7060, Tokyo, Japan).

### Body composition

Subjects underwent body composition measurements before and after each phase of the intervention. All subjects were required to fast for 8 hours before the measurement. During the test, all subjects stood on the bottom electrode with arms extended at a 30° angle to the torso, held the induction handle with both hands, and did not move or speak. Measurements were performed within 60 s at 1, 5, 50, 260, 500, and 1000 kHz using a bioelectrical impedance analyzer (BIA) (InBody 570, In-body, Seoul, Korea) with the multi-frequency principle.

### Clinical biochemistry and hematology analysis

Blood was collected from each subject before each phase of supplementation for clinical biochemical and hematological analysis to confirm the basic biochemical indicators and health status. All the subjects were asked to fast for 8 hours the night before blood was drawn. After blood collection, the serum was obtained by centrifugation, and aspartate transaminase (AST), alanine aminotransferase (ALT), albumin (ALB), blood urea nitrogen (BUN), creatinine (CREA), uric acid (UA), total protein (TP), total cholesterol (TC), triglyceride (TG), high-density lipoprotein (HDL), and low-density lipoprotein (LDL) indicators were measured using an automatic analyzer (Hitachi 7060, Hitachi, Tokyo, Japan). In addition, the complete blood count (CBC) profiles were also analyzed (MindrayBC-2800 Vet, Shenzhen, China).

### Statistical analysis

All data are expressed as the mean ± SD. Statistical analyses were performed in IBM SPSS Statistics ver. 24.1 (IBM Co., Armonk, NY, USA). Differences within groups before and after the intervention were analyzed using a Bonferroni-adjusted paired *t*-test. Intergroup differences were analyzed using Student's unpaired *t*-test. For non-parametric data, including fat mass and ratio of body composition change, the Mann-Whitney *U* test was used for comparison. Time to exhaustion was analyzed via two-way repeated-measures ANOVA with post hoc Bonferroni test. Differences were considered statistically significant at *p* < 0.05. We used the Harvard calculator (http://hedwig.mgh.harvard.edu/sample_size/size.html) to calculate the required sample size for the clinical trial. We assumed a 0.05 significance level, a power of 0.8, and a standard deviation of the difference within 0.73. From these assumptions, at least 32 patients were required for this two-treatment crossover study.

## Results

### Subjects' dietary analysis and Body composition changes

In order to ensure that subjects' exercise performance and fatigue biochemical values were not affected by differences in dietary intake and energy, 3 days dietary record analysis was performed before and after Ex PLUS^®^ supplementation. As shown in **Table 2**, there were no significant differences in carbohydrate, protein, or fat intake between the placebo and Ex PLUS^®^ groups before and after the intervention, and there were no significant changes within either group after the intervention. On the other hand, body composition can also be affected by dietary changes, which, in turn, can affect exercise performance. The Ex PLUS^®^ supplementation did not cause changes in body composition, nor was there a significant difference between the two groups (**Table 2**).

### Effects of Ex PLUS® supplementation on biochemical parameters and hematology

Table 3 shows the routine serum biochemical indicators and hematological data of the placebo and Ex PLUS^®^ groups before and after the intervention. The results showed that all subjects participated in this trial in a healthy state, and there was no damage to various indicators in the blood after the intervention.

### Effects of Ex PLUS® supplementation on fatigue biochemical parameters during exercise and rest

**Figure 3:**
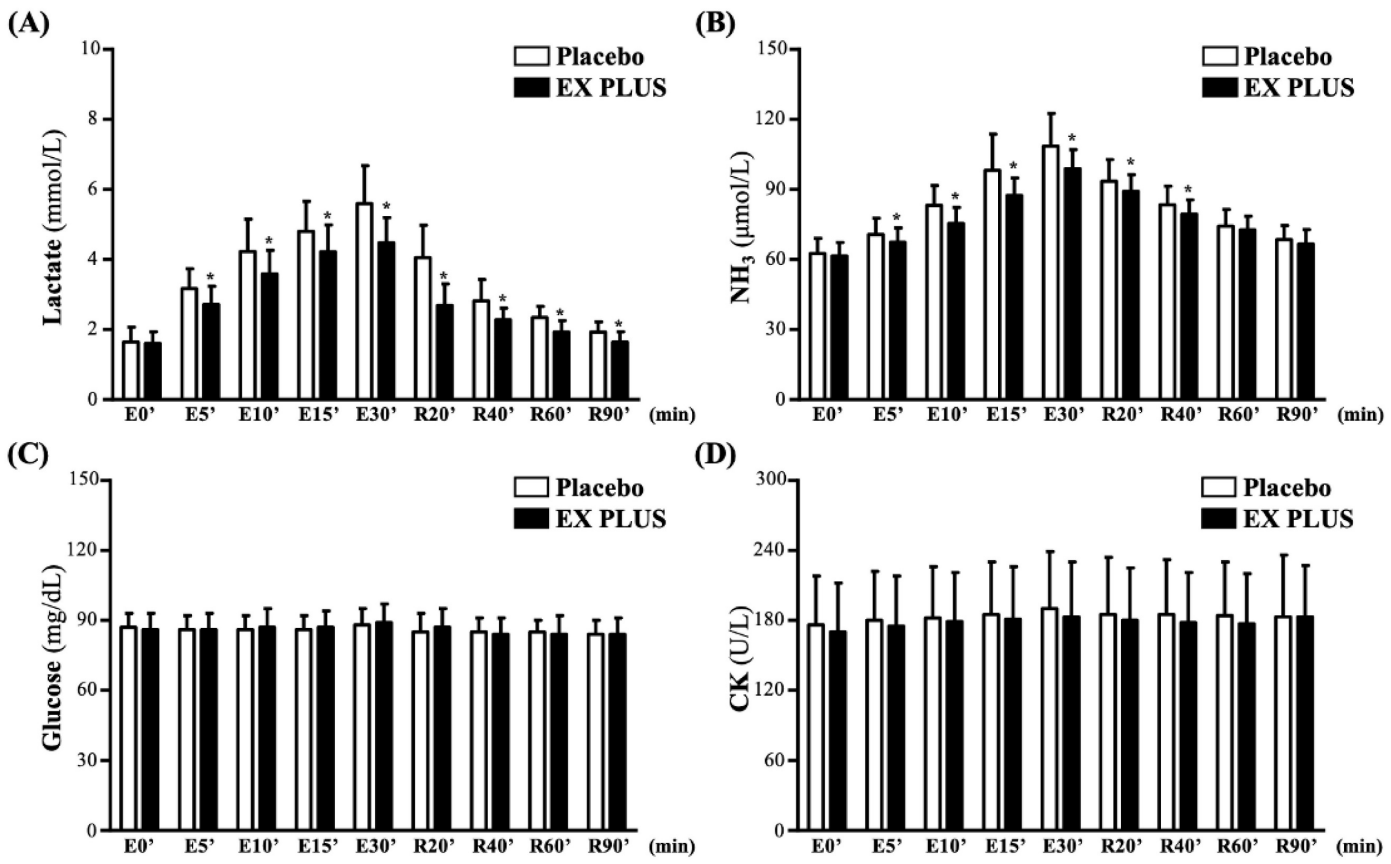
Effects of Ex PLUS^®^ supplementation on (A) lactate, (B) NH3, (C) CK, and (D) glucose serum levels during and after exercise. Data are shown as the mean ± SD. *Significant difference compared to the placebo group (*p*<0.05).

Both lactate and NH3 levels gradually increased during exercise and gradually decreased after rest and recovery. As shown in **Figure 3A**, compared to the placebo group, the Ex PLUS^®^ group presented a significantly reduced level of lactate production from exercise at 5 minutes until 30 minutes after exercise, with accelerated recovery from rest at 20 minutes until test completion (*p <* 0.05). This was similar to the NH3 levels, with the Ex PLUS^®^ group presenting a significantly reduced NH3 production level from 5 minutes to 30 minutes after exercise and accelerated recovery from rest at 20 and 40 minutes compared to the placebo group (*p <* 0.05) (**Figure 3B**). However, during recovery from exercise and rest, the glucose level and CK activity did not fluctuate significantly, and there was no significant difference in them between the two groups (**Figure 3C and 3D**).

### Effects of Ex PLUS® supplementation on endurance performance

**Figure 4:**
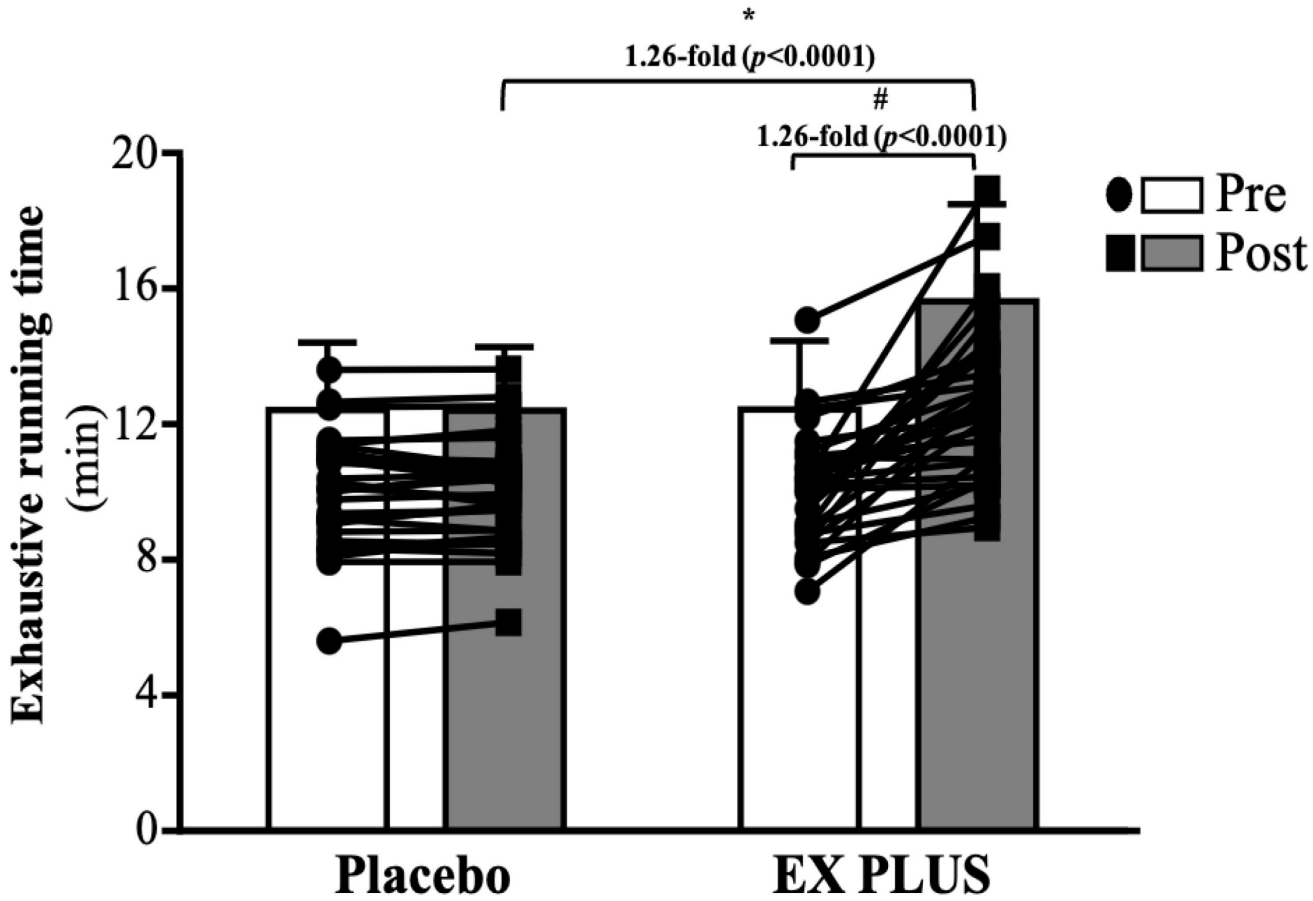
Effects of Ex PLUS^®^ supplementation on the time to exhaustion. Data are shown as the mean ± SD, n = 32 subjects/group. *Significant difference compared to the placebo group (*p*<0.05). #Significant difference (*p*<0.05) compared with the pretest in the same group.

Before intervention, the exhaustion test times were 12.57 ± 2.03 and 12.59 ± 2.06 (min) for the placebo and Ex PLUS^®^ groups, respectively. There was no significant difference between the two groups. After 28 days of intervention, the times to exhaustion were 12.56 ± 1.90 and 15.82 ± 1.91 (min) for the placebo and Ex PLUS^®^ groups, respectively. The Ex PLUS^®^ group showed a significant 1.26-fold improvement in time to exhaustion with regard to both baseline measurements (*p <* 0.0001) and the placebo group (*p <* 0.0001) (**Figure 4**).

## Discussion

In humans, nutrients and energy are primarily used by the body through food intake. Although the accompanying vitamins or minerals do not act as a primary energy source, they play a vital role in energy metabolism. In the current study, we found that consecutive 28 days of Ex PLUS^®^ supplementation improved exercise endurance performance and reduced fatigue metabolites, such as lactate or blood ammonia in not athletes. In addition, supplementation in appropriate doses did not cause adverse effects on the human body.

B vitamins are involved in the regulation of energy metabolism and in the synthesis and degradation of carbohydrates, proteins, and fats 24. Moderate supplementation has potential synergistic effects on improving physical activity, energy metabolism, and exercise performance 25. The accumulation of lactate and ammonia during exercise is generally believed to increase hydrogen ions and acidosis in muscles and is considered a major cause of muscle fatigue 26. Thiamine is primarily stored in muscles, and when the intracellular concentration of thiamine decreases, it reduces the activation of enzymes that lead to ATP biosynthesis; this causes fatigue, which, in turn, reduces exercise performance 27. However, an increase in serum thiamine is associated with a decrease in pyruvate and lactate levels 28. A past animal study observed a decrease in lactate production following thiamine administration 29. In addition, in trials of three crossover treatments (placebo, training, and thiamine), both training and thiamine intervention reduced blood lactate concentrations in athletes during exercise 30. This might be due to the fact that thiamine not only is a coenzyme of pyruvate dehydrogenase, which stimulates pyruvate, but also can be converted to acetyl-CoA by the decarboxylation of BCAA and α-keto acids, which then enter the citric acid cycle to generate NADH and FADH2 and then generate ATP through the electron transport chain to provide the energy required for movement 2, 31, 32. In our previous animal experiments, we found that 4 weeks of thiamine tetrahydrofurfuryl disulfide supplementation significantly improved the endurance performance of mice, showing a dose-dependent effect 13. The current study also confirmed that vitamin B complex supplementation in humans could reduce the rate of increase in lactate during fixed-intensity exercise and had the effect of accelerating clearance (**Figure 3A**). On the other hand, riboflavin is composed of two coenzymes (FAD and FMN) and is an important component involved in the energy metabolism of carbohydrates, fats, and proteins and in electron transfer in steroid hormone production 32. Among them, FAD is involved in further energy production through the β-oxidation of fatty acids and through the oxidative decarboxylation of pyruvate to produce acetyl-CoA from the catabolism of glucose and branched-chain amino acids 9.

Among other B vitamins, vitamin B6, folic acid, and vitamin B12 all contribute to the metabolism of homocysteine and are involved in the metabolism of proteins and amino acids, which, in turn, play a role in important pathways used during physical activity 33. Among them, folic acid and vitamin B12 are also key nutrients for damaged cell and tissue repair as coenzymes for deoxyribonucleic acid (DNA) synthesis, erythrocyte synthesis, amino acid metabolism, and the decomposition of odd fatty acid chains, respectively 34. In addition, vitamin B12 acts as a cofactor to convert amino acids to propionyl-CoA, while fatty acids are oxidized to acetyl-CoA and propionyl-CoA. After entering the tricarboxylic acid (TCA) cycle, propionyl-CoA is carboxylated to methylmalonyl-CoA and finally converted to succinyl-CoA by methylmalonyl-CoA mutase to generate energy 35. Pyridoxal 5'-phosphate (PLP) is the biologically active form of vitamin B6 in the human body, mainly involved in the metabolism of proteins and amino acids, and is a cofactor for transaminases, decarboxylases, and other enzymes for the metabolic transformation of amino acids and nitrogenous compounds 25, 36, 37. Ammonia has long been recognized as a central and peripheral factor in the onset of exercise-induced fatigue, and its level increases with exercise intensity and duration 38, 39. Despite many theories, there have been few studies on B vitamins reducing blood ammonia levels after exercise. In a past study, administration of BCAAs supplemented with vitamin B6 for 8 weeks in taekwondo athletes was found to reduce post-exercise NH3 concentrations 40. Although our study did not include the addition of protein supplements, vitamin B complex supplementation alone significantly reduced the increase in blood ammonia levels during exercise (**Figure 3B**). However, the lower increase in NH_3_ concentration after exercise may be associated with lower ATP consumption and slower rates of glycolysis and glycogenolysis 41. Another way that vitamin B6 produces energy is by participating in glycogenolysis and gluconeogenesis during exercise 42. During exercise, the process of gluconeogenesis involves the breakdown of amino acids to provide energy to muscles and the conversion of lactate to glucose in the liver, and several enzymes are involved in this metabolically driven conversion 43. This may be one of the factors that improved exercise endurance performance in this study. Additionally, a past study reported on eight physically active subjects who performed two tests on a bicycle ergometer at 60% and 85% of VO_2max_ for 30 minutes and 20 minutes, respectively. The results found no significant difference in blood glucose concentration 44 which was similar to our findings (**Figure 3D**), but the related mechanism still needs to be further explored.

In the current study, we demonstrated that consecutive 28 days of vitamin B complex supplementation significantly improved exercise endurance performance. In addition to helping the metabolism of nutrients to produce energy through the B vitamins, other elements may be involved. Taurine is an important free amino acid that, although not fully involved in protein synthesis, has multiple physiological effects, including regulation of osmotic pressure, membrane stability and calcium kinetics, as well as the enhancement of systemic anti-inflammatory responses and total antioxidant capacity 45, 46. These properties can directly boost physical performance and reduce muscle fatigue. In addition, other studies have shown that taurine enhances exercise endurance performance by enhancing the gluconeogenesis of amino acids (AAs) in the liver and levels of glycerol in the blood, promoting lipid metabolism, and improving hepatic glucose storage and energy 47. On the other hand, taurine deficiency increases the NADH/NAD^+^ ratio, inhibits pyruvate dehydrogenase activity, and causes pyruvate deficiency, thereby significantly reducing glucose oxidation and ATP biosynthesis 48, which may affect CK levels in blood and muscle 45. A past study showed that acute taurine supplementation (approximately 1 g taken 5 times daily before and after exercise) during endurance training was effective in reducing blood lactate levels during endurance training 49. However, our dose was only 2% of that used in the past study, so the reduction in lactate concentrations in the current study may not be entirely the benefit of taurine supplementation. Furthermore, CK activity did not change significantly in this study, which may be due to the fact that CK activity usually increases gradually 2 hours after exercise and peaks at 24-72 hours, which may also be affected by exercise intensity 50. These may be factors that contributed to the relative flattening of CK activity in the blood during and after exercise in this study (**Figure 3C**). Unlike the B vitamins, vitamin E acts as a fat-soluble and chain-breaking antioxidant that prevents the progression of lipid peroxidative chain reactions and maintains the integrity of polyunsaturated fatty acids in cell membranes 51. Although vitamin E plays a key role in protecting the central nervous system from free radical damage, there is still a polar debate about whether vitamin E protects against post-exercise injury 52. Additionally, vitamin E was not found to interact with B vitamins in an earlier study 53. Therefore, it is necessary to further explore whether the combination of vitamin E and B vitamins has a better synergistic effect on exercise performance in the future.

In the current study, we demonstrated that B-complex vitamin supplementation (Ex PLUS^®^) has the effect of improving exercise endurance performance and reducing exercise fatigue. However, there are still some limitations in the research process, including the following: 1: There are congenital differences in physique or physiological metabolism between men and women. To more clearly explore the efficacy of placebo and Ex PLUS^®^ in improving exercise performance and reducing fatigue, a gender-excluded statistical analysis was performed. 2: In this study, subjects were required to provide blood samples at different time points during exercise and at rest after exercise. After analyzing the indicators related to exercise fatigue, there was no extra blood to analyze the concentrations of various B vitamins in the blood. 3: The importance of glycogen storage for exercise endurance performance is well known. However, in human trials, muscle puncture is required to detect glycogen levels in muscle. This may involve regulations in medical practice and the need for subjects to recover over an extended period of time, thus excluding glycogen testing from this study.

## Conclusions

In conclusion, in the current study, we demonstrated that 28 consecutive days of supplementation with Ex PLUS^®^ (with a vitamin B complex, taurine, and vitamin E) increased exercise endurance performance, reduced post-exercise fatigue metabolite production, and accelerated recovery in not athletes. In addition, adequate supplementation does not cause any adverse damage or burden to the human body.

## Figures and Tables

**Table 1:** Subjects' basic information.

Characteristics	Placebo in stage I	Ex PLUS^®^ in stage I
Age (years)	21.3±1.2 ^a^	21.3±1.2 ^a^
Height (cm)	168.8±8.9 ^a^	168.8±8.9 ^a^
Weight (kg)	62.4±7.6 ^a^	63.5±7.2 ^a^
VO_2max_ (mL/kg/min)	44.5±5.1 ^a^	44.3±5.0 ^a^

Data are presented as mean ± SD. The same superscript letters (a) indicate no significant difference between groups at *p* > 0.05.

**Table 2:** Subjects' dietary intake and body composition.

Characteristics	Items	Before		After	
		Placebo	Ex PLUS^®^	Placebo	Ex PLUS^®^
Dietary intake	Carbohydrate (g/day)	172±27 ^a^	171±19 ^a^	169±24 ^a^	167±18 ^a^
	Protein (g/day)	71±19 ^a^	70±14 ^a^	69±18 ^a^	70±17 ^a^
	Fat (g/day)	75±16 ^a^	74±8 ^a^	77±13 ^a^	76±8 ^a^
	Total Calories (kcal/day)	1654±272 ^a^	1628±140 ^a^	1648±257 ^a^	1627±130 ^a^
Body composition	Body weight (kg)	62.4±7.6 ^a^	63.5±7.2 ^a^	62.3±7.9 ^a^	62.9±7.1 ^a^
	BMI (kg/m^2^)	21.9±1.9 ^a^	22.3±2.0 ^a^	21.9±1.6 ^a^	22.1±1.6 ^a^
	Muscle mass (kg)	28.3±5.3 ^a^	28.4±5.3 ^a^	28.2±5.4 ^a^	28.2±5.2 ^a^
	Fat mass (%)	19.4±6.7 ^a^	20.6±6.9 ^a^	19.4±6.8 ^a^	20.5±7.0 ^a^

Data are presented as mean ± SD. The same superscript letters (a) indicate no significant difference between groups at *p* > 0.05. BMI, body mass index.

**Table 3:** Biochemical analysis and blood count profiles of the subjects before and after the intervention.

Characteristics	Items	Before		After	
		Placebo	Ex PLUS^®^	Placebo	Ex PLUS^®^
Biochemistry parameters	AST (U/L)	24±5 ^a^	23±5 ^a^	24±4 ^a^	23±5 ^a^
	ALT (U/L)	14±4 ^a^	14±3 ^a^	14±4 ^a^	14±3 ^a^
	ALB (g/dL)	5.15±0.33 ^a^	5.15±0.30 ^a^	5.20±0.24 ^a^	5.17±0.30 ^a^
	TC (mg/dL)	191±20 ^a^	193±25 ^a^	193±20 ^a^	193±25 ^a^
	TG (mg/dL)	54±14 ^a^	57±16 ^a^	57±13 ^a^	57±16 ^a^
	HDL (mg/dL)	67.6±6.2 ^a^	67.2±6.2 ^a^	68.3±5.8 ^a^	67.5±5.5 ^a^
	LDL (mg/dL)	94.6±12.6 ^a^	95.2±12.6 ^a^	95.6±12.4 ^a^	95.6±12.2 ^a^
	BUN (mg/dL)	15.7±2.6 ^a^	15.3±2.7 ^a^	15.8±2.4 ^a^	15.4±2.8 ^a^
	CREA (g/dL)	1.10±0.11 ^a^	1.08±0.12 ^a^	1.11±0.10 ^a^	1.09±0.12 ^a^
	UA (mg/dL)	5.2±1.0 ^a^	5.2±1.1 ^a^	5.3±1.0 ^a^	5.2±1.1 ^a^
	Glucose (mg/dL)	81±6 ^a^	83±7 ^a^	82±6 ^a^	83±7 ^a^
CBC	WBCs (10^3^/mcL)	5.9±0.9 ^a^	5.9±0.8 ^a^	6.1±0.8 ^a^	6.2±0.8 ^a^
	Neutrophils (%)	54.7±5.3 ^a^	54.1±6.7 ^a^	54.5±6.2 ^a^	53.9±8.9 ^a^
	Lymphocytes (%)	34.2±4.4 ^a^	34.7±6.6 ^a^	35.0±5.1 ^a^	35.3±8.7 ^a^
	Monocytes (%)	7.5±1.4 ^a^	7.3±1.4 ^a^	7.0±1.7 ^a^	7.0±1.5 ^a^
	Eosinophils (%)	3.0±1.8 ^a^	3.0±2.2 ^a^	3.2±2.0 ^a^	2.7±1.9 ^a^
	Basophils (%)	0.7±0.2 ^a^	0.8±0.4 ^a^	0.7±0.3 ^a^	0.8±0.4 ^a^
	Platelets (10^3^/mcL)	260±33 ^a^	262±36 ^a^	251±44 ^a^	255±39 ^a^

Data are shown as the mean ± SD. Statistical significance between the Placebo and Ex PLUS^®^ groups at the same collection time points was analyzed using Student's unpaired t-test. The same superscript letter (a) indicates no significant differences among the groups at *p* > 0.05. AST, aspartate aminotransferase; ALT, alanine aminotransferase; BUN, blood urea nitrogen; CREA, creatine; UA, uric acid; TP, total protein; TC, total cholesterol; TG, triacylglycerol. HDL, high-density lipoprotein; LDL, low-density lipoprotein; WBC, white blood cell.
